# Supplementary material for: Exploring the Antibiotic Resistance Profile of Clinical Klebsiella pneumoniae Isolates in Portugal
Source: Antibiotics (Basel). 2022 Nov 13;11(11):1613. doi: 10.3390/antibiotics11111613 (PMC9686965; doi:10.3390/antibiotics11111613)
Supplement: Supplementary file 1 [file antibiotics-11-01613-s001.zip › Table S1.pdf]

Isolate no.	Source sample	Year	Doripenem (DOR)	Ertapenem (ERT)	Meropenem (MER)	Imipenem (IMI)	Amoxicillin-clavulanic acid (AMC)	Ampicillin-sulbactam (AMS)	Piperacillin-tazobactam (PIT)	Ticarcillin-clavulanic acid (TIL)	Aztreonam (AZT)	Cefoxitin (CXI)	Cefotetan (CTT)	Cefuroxime (CUR)	Cefazolin (CZO)	Cefotaxime (CTA)	Ceftazidime (CTZ)	Cefepime (CEP)	Ceftaroline (CTL)	Resistance phenotype	Resistance genes
H51	Urine	2010	S	R	I	S	R	R	S	R	R	R	S	R	R	R	R	I	R	ESBL and/or AmpC	blaSHV; blaCMY-2
H52	Urine	2010	S	S	S	S	R	R	S	R	R	S	S	R	R	R	R	R	R	ESBL	blaTEM; blaSHV; blaCTX-M
H53	Urine	2010	S	S	S	S	R	R	R	R	R	S	S	R	R	R	R	R	R	CPN and/or ESBL	blaKPC; blaTEM; blaSHV; blaCTX-M
H54	Urine	2010	S	R	S	S	R	R	S	R	I	R	R	R	R	R	R	R	R	CPN, ESBL and/or AmpC	blaKPC; blaSHV; blaCMY-2
H55	Urine	2010	S	S	S	S	S	S	S	S	S	S	S	R	R	R	R	R	R	CPN and/or ESBL	blaKPC; blaSHV
H56	Sputum	2010	S	S	S	S	R	R	S	R	R	S	S	R	R	R	R	R	R	CPN and/or ESBL	blaKPC; blaTEM; blaSHV; blaCTX-M
H57	Blood culture	2010	S	R	I	S	R	R	R	R	R	S	S	R	R	R	R	R	R	CPN and/or ESBL	blaKPC; blaTEM; blaSHV; blaCTX-M
H58	Urine	2010	S	R	I	S	R	R	R	R	R	R	S	R	R	R	R	R	R	CPN, ESBL and/or AmpC	blaKPC; blaTEM; blaSHV; blaCTX-M
H59	Skin exudate	2010	S	R	S	S	R	R	S	R	R	R	S	R	R	R	R	I	R	ESBL and/or AmpC	blaSHV; blaCMY-2
H60	Urine	2010	S	S	S	S	R	R	R	R	R	S	S	R	R	R	R	R	R	ESBL	blaTEM; blaSHV; blaCTX-M
H61	Sputum	2010	S	R	S	S	R	R	R	R	I	R	R	R	R	R	R	R	R	CPN, ESBL and/or AmpC	blaKPC; blaSHV; blaCMY-2
H62	Urine	2010	S	R	S	S	R	R	R	R	R	R	S	R	R	R	R	R	R	CPN, ESBL and/or AmpC	blaKPC; blaTEM; blaSHV; blaCTX-M
H63	Catheter tip	2010	S	S	S	S	R	R	R	R	R	R	S	R	R	R	R	R	R	CPN, ESBL and/or AmpC	blaKPC; blaTEM; blaSHV; blaCTX-M
H64	Urine	2010	S	S	S	S	R	R	R	R	R	R	S	R	R	R	R	R	R	ESBL and/or AmpC	blaKPC; blaTEM; blaSHV; blaCTX-M
H65	Urine	2010	S	S	S	S	R	R	R	R	R	S	S	R	R	R	R	R	R	CPN and/or ESBL	blaKPC; blaTEM; blaSHV; blaCTX-M
H66	Urine	2014	S	R	S	S	R	R	R	R	I	R	R	R	R	R	R	I	R	ESBL and/or AmpC	blaSHV; blaDHA
H67	Blood culture	2014	S	R	S	S	R	R	R	R	R	R	S	R	R	R	R	R	R	ESBL and/or AmpC	blaSHV; blaCTX-M
H68	Sputum	2014	S	R	S	S	R	R	R	R	R	R	I	R	R	R	R	R	R	ESBL and/or AmpC	blaTEM; blaSHV; blaCTX-M; blaDHA
H69	Urine	2014	R	R	R	R	R	R	R	R	R	R	I	R	R	R	R	R	R	CPN, ESBL and/or AmpC	blaKPC; blaTEM
H70	Sputum	2014	R	R	R	I	R	R	R	R	R	R	S	R	R	R	R	R	R	CPN, ESBL and/or AmpC	blaKPC; blaTEM; blaSHV
H71	Sputum	2017	R	R	I	R	R	R	R	R	R	R	R	R	R	R	R	R	R	CPN, ESBL and/or AmpC	blaKPC; blaSHV
H72	Blood culture	2017	S	S	S	S	R	R	R	R	R	S	S	R	R	R	R	R	R	ESBL	blaTEM; blaSHV; blaCTX-M
H73	Pus	2017	S	S	S	S	R	R	R	R	R	R	S	R	R	R	R	R	R	ESBL and/or AmpC	blaTEM; blaSHV; blaCTX-M
H74	Skin exudate	2017	S	S	S	S	R	R	R	R	R	R	S	R	R	R	R	R	R	ESBL and/or AmpC	blaTEM; blaSHV; blaCTX-M
H75	Sputum	2017	S	S	S	S	R	R	R	R	R	S	S	R	R	R	R	R	R	ESBL	blaTEM; blaSHV; blaCTX-M
H76	Bronchoalveolar lavage	2017	S	S	S	S	R	R	R	R	R	S	S	R	R	R	R	R	R	ESBL	blaTEM; blaSHV; blaCTX-M
H77	Sputum	2017	S	S	S	S	R	R	R	R	R	S	S	R	R	R	R	R	R	ESBL	blaTEM; blaSHV; blaCTX-M
H78	Skin exudate	2017	S	S	S	S	R	R	R	R	R	S	S	R	R	R	R	R	R	ESBL	blaTEM
H79	Blood culture	2017	S	S	S	S	R	R	R	R	R	S	S	R	R	R	R	R	R	ESBL	blaTEM; blaSHV; blaCTX-M
H80	Pus	2017	S	S	S	S	R	R	R	R	I	R	R	R	R	R	R	S	R	ESBL and/or AmpC	blaSHV; blaDHA
H81	Bronchoalveolar lavage	2017	S	S	S	S	R	R	R	R	R	S	S	R	R	R	R	R	R	ESBL	blaSHV; blaCTX-M
H82	Pus	2019	R	R	R	I	R	R	R	R	R	R	R	R	R	R	R	R	R	CPN and/or ESBL	blaKPC; blaSHV
H83	Surgical wound	2019	R	R	R	R	R	R	R	R	R	R	S	R	R	R	R	R	R	CPN, ESBL and/or AmpC	blaKPC; blaSHV
H84	Bronchial aspirate	2019	R	R	I	I	R	R	R	R	R	S	S	R	R	R	R	R	R	CPN and/or ESBL	blaKPC; blaTEM; blaSHV
H85	Urine	2019	R	R	R	R	R	R	R	R	R	R	S	R	R	R	R	R	R	CPN, ESBL and/or AmpC	blaKPC
H86	Sputum	2019	S	S	S	S	R	R	R	R	R	R	S	R	R	R	R	R	R	ESBL and/or AmpC	blaTEM; blaSHV; blaCTX-M
H87	Blood culture	2019	R	R	R	R	R	R	R	R	R	R	R	R	R	R	R	R	R	CPN, ESBL and/or AmpC	blaKPC; blaTEM; blaSHV; blaCTX-M
H88	Urine	2019	R	R	R	I	R	R	R	R	R	S	I	R	R	R	R	R	R	CPN and/or ESBL	blaKPC; blaSHV
H89	Urine	2019	R	R	R	R	R	R	R	R	R	R	R	R	R	R	R	R	R	CPN, ESBL and/or AmpC	blaKPC; blaSHV
H90	Urine	2019	R	R	R	R	R	R	R	R	R	R	S	R	R	R	R	R	R	CPN, ESBL and/or AmpC	blaKPC; blaTEM; blaSHV; blaCTX-M
H91	Blood culture	2019	R	R	R	R	R	R	R	R	R	S	S	R	R	R	R	R	R	CPN and/or ESBL	blaKPC; blaTEM; blaSHV
H92	Blood culture	2019	S	S	S	S	R	R	R	R	R	S	S	R	R	R	R	R	R	ESBL	-
H93	Blood culture	2019	R	R	R	R	R	R	R	R	R	R	I	R	R	R	R	R	R	CPN, ESBL and/or AmpC	blaKPC; blaSHV
H94	Rectal swab	2019	I	R	R	I	R	R	R	R	R	S	I	R	R	R	R	R	R	CPN and/or ESBL	blaTEM; blaSHV; blaCTX-M
H95	Catheter tip	2019	R	R	R	R	R	R	R	R	R	R	S	R	R	R	R	R	R	CPN, ESBL and/or AmpC	blaKPC
H96	Urine	2019	R	R	R	S	R	R	R	S	R	S	S	R	S	R	R	R	S	CPN and/or ESBL	blaKPC; blaTEM; blaCTX-M
H97	Urine	2019	R	R	R	R	R	R	R	R	R	R	I	R	R	R	R	R	R	CPN, ESBL and/or AmpC	blaKPC
H98	Urine	2019	R	R	R	R	R	R	R	R	R	R	R	R	R	R	R	R	R	CPN, ESBL and/or AmpC	blaKPC; blaTEM; blaSHV; blaCTX-M
H99	Other products	2019	I	R	S	I	R	R	R	R	R	S	S	R	R	R	R	R	R	CPN and/or ESBL	blaKPC; blaSHV
H100	Bronchial aspirate	2019	R	R	R	I	R	R	R	R	R	R	I	R	R	R	R	R	R	CPN, ESBL and/or AmpC	blaKPC; blaSHV
H101	Peritoneal fluid	2020	R	R	R	R	R	R	R	R	R	R	R	R	R	R	R	R	R	CPN, ESBL and/or AmpC	blaTEM; blaSHV; blaCTX-M
H102	Urine	2020	R	R	I	I	R	R	R	R	R	R	R	R	R	R	R	R	R	CPN, ESBL and/or AmpC	blaKPC; blaTEM; blaSHV; blaCTX-M
H103	Urine	2020	R	R	R	S	R	R	R	R	R	R	I	R	R	R	R	R	R	CPN, ESBL and/or AmpC	blaOXA-48; blaTEM; blaSHV; blaCTX-M; blaDHA
H104	Other products	2018	R	R	I	S	R	R	R	R	R	R	I	R	R	R	R	R	R	CPN, ESBL and/or AmpC	blaTEM; blaSHV; blaCTX-M
MJH 537	Rectal swab	2018	R	R	R	R	R	R	R	R	R	R	S	R	R	R	R	R	R	CPN, ESBL and/or AmpC	blaKPC; blaTEM; blaSHV; blaCTX-M
MJH 538	Rectal swab	2018	R	R	R	R	R	R	R	R	R	R	I	R	R	R	R	R	R	CPN, ESBL and/or AmpC	blaKPC; blaSHV
MJH 539	Rectal swab	2018	R	R	R	R	R	R	R	R	R	S	S	R	R	R	R	R	R	CPN and/or ESBL	blaKPC; blaSHV
MJH 540	Rectal swab	2018	R	R	R	R	R	R	R	R	R	R	S	R	R	R	R	R	R	CPN, ESBL and/or AmpC	blaKPC; blaSHV
MJH 541	Rectal swab	2018	R	R	I	I	R	R	R	R	R	S	S	R	R	R	R	R	R	CPN and/or ESBL	blaKPC
MJH 542	Urine	2018	R	R	I	I	R	R	R	R	R	S	R	R	R	R	R	R	R	CPN and/or ESBL	blaOXA-48; blaSHV; blaCTX-M
MJH 543	Urine	2018	R	R	R	R	R	R	R	R	R	R	S	R	R	R	R	R	R	CPN, ESBL and/or AmpC	blaKPC; blaSHV
MJH 544	Urine	2018	R	R	R	R	R	R	R	R	R	S	R	R	R	R	R	R	R	CPN and/or ESBL	blaKPC; blaSHV
MJH 545	Percutaneous drainage of liver abscess	2018	R	S	S	I	R	R	R	R	R	S	S	R	R	R	R	R	R	CPN and/or ESBL	blaKPC; blaTEM; blaSHV
MJH 546	Urine	2018	R	R	I	I	R	R	R	R	R	S	S	R	R	R	R	R	R	CPN and/or ESBL	blaKPC; blaTEM; blaSHV
MJH 548	Catheter tip	2018	R	R	R	R	R	R	R	R	R	R	R	R	R	R	R	R	R	CPN, ESBL and/or AmpC	blaKPC; blaSHV
MJH 549	Bronchial aspirate	2018	R	R	I	R	R	R	R	R	R	R	S	R	R	R	R	R	R	CPN, ESBL and/or AmpC	blaKPC; blaTEM; blaSHV
MJH 550	Blood culture	2018	S	S	S	I	R	R	R	I	R	S	S	R	R	R	R	R	R	CPN and/or ESBL	blaTEM; blaSHV; blaCTX-M

MJH 551	Urine	2018	R	R	I	R	R	R	R	R	R	R	S	R	R	R	R	R	R	CPN, ESBL and/or AmpC	blaKPC; blaTEM; blaSHV
MJH 552	Sputum	2018	R	R	I	I	R	R	R	R	R	R	S	R	R	R	R	R	R	CPN, ESBL and/or AmpC	blaKPC; blaTEM; blaSHV
MJH 553	Ulcer biopsy after pretibial trauma	2018	R	R	I	I	R	R	R	R	R	S	S	R	R	R	R	R	R	CPN and/or ESBL	blaKPC; blaTEM; blaSHV
MJH 554	Urine	2018	R	R	R	R	R	R	R	R	R	R	S	R	R	R	R	R	R	CPN, ESBL and/or AmpC	blaKPC; blaSHV
MJH 555	Rectal swab	2018	R	R	R	I	R	R	R	R	R	R	I	R	R	R	R	R	R	CPN, ESBL and/or AmpC	blaKPC; blaTEM; blaSHV
MJH 557	Rectal swab	2018	R	R	R	R	R	R	R	R	R	R	R	R	R	R	R	R	R	CPN, ESBL and/or AmpC	blaKPC; blaSHV
MJH 558	Rectal swab	2018	R	R	R	R	R	R	R	R	R	S	S	R	R	R	R	R	R	CPN and/or ESBL	blaKPC; blaSHV
MJH 560	Rectal swab	2018	R	R	R	R	R	R	R	R	R	R	S	R	R	R	R	R	R	CPN, ESBL and/or AmpC	blaKPC; blaSHV
MJH 561	Urine	2018	R	R	R	I	R	R	R	R	R	R	S	R	R	R	R	R	R	CPN, ESBL and/or AmpC	blaSHV; blaCTX-M
MJH 562	Urine	2018	R	R	I	I	R	R	R	R	R	R	S	R	R	R	R	R	R	CPN, ESBL and/or AmpC	blaKPC; blaSHV; blaCTX-M
MJH 563	Rectal swab	2018	R	R	I	R	R	R	R	R	R	R	I	R	R	R	R	R	R	CPN, ESBL and/or AmpC	blaKPC
MJH 564	Urine	2018	R	R	I	I	R	R	R	R	R	R	S	R	R	R	R	R	R	CPN, ESBL and/or AmpC	blaKPC; blaTEM
MJH 566	Rectal swab	2018	R	R	R	R	R	R	R	R	R	S	S	R	R	R	R	R	R	CPN and/or ESBL	blaKPC; blaTEM; blaSHV
MJH 567	Rectal swab	2018	S	R	S	I	R	R	R	R	R	S	S	R	R	R	R	R	R	CPN and/or ESBL	blaKPC; blaSHV
MJH 569	Urine	2018	R	R	I	R	R	R	R	R	R	R	I	R	R	R	R	R	R	CPN, ESBL and/or AmpC	blaKPC; blaSHV
MJH 570 1F	Blood culture	2018	R	R	I	R	R	R	R	R	R	R	S	R	R	R	R	R	R	CPN, ESBL and/or AmpC	blaKPC; blaSHV
MJH 570 2F	Blood culture	2018	R	R	I	R	R	R	R	R	R	S	S	R	R	R	R	R	R	CPN and/or ESBL	blaKPC
MJH 571	Rectal swab	2018	R	R	I	R	R	R	R	R	R	R	S	R	R	R	R	R	R	CPN, ESBL and/or AmpC	blaKPC
MJH 599	Bronchial aspirate	2018	R	R	I	I	R	R	R	R	R	S	S	R	R	R	R	R	R	CPN and/or ESBL	blaOXA-48; blaCTX-M
MJH 602	Retroperitoneal drain of necrotizing pancreatitis	2018	R	R	I	I	R	R	R	R	R	S	S	R	R	R	R	R	R	CPN and/or ESBL	blaKPC
MJH 604	Urine	2018	R	R	I	R	R	R	R	R	R	R	S	R	R	R	R	R	R	CPN, ESBL and/or AmpC	blaKPC; blaTEM; blaSHV
MJH 606	Blood culture	2018	R	R	I	R	R	R	R	R	R	R	S	R	R	R	R	R	R	CPN, ESBL and/or AmpC	blaKPC
MJH 607	Urine	2018	R	R	I	I	R	R	R	R	R	R	S	R	R	R	R	R	R	CPN, ESBL and/or AmpC	blaKPC
MJH 608	Urine	2018	R	R	R	R	R	R	R	R	R	S	S	R	R	R	R	R	R	CPN and/or ESBL	blaKPC
MJH609	Urine	2018	R	R	R	R	R	R	R	R	R	R	R	R	R	R	R	R	R	CPN, ESBL and/or AmpC	blaKPC; blaCTX-M
MJH 610	Urine	2018	I	S	I	I	R	R	R	R	R	S	S	R	R	I	R	R	R	CPN and/or ESBL	blaKPC
MJH 612	Urine	2018	R	R	S	I	R	R	R	R	R	R	S	R	R	R	R	R	R	CPN, ESBL and/or AmpC	blaKPC; blaTEM; blaCTX-M
MJH 613	Blood culture	2018	R	R	I	R	R	R	R	R	R	R	I	R	R	R	R	R	R	CPN, ESBL and/or AmpC	blaKPC
MJH 614	Bronchial aspirate	2018	R	R	R	R	R	R	R	R	R	R	I	R	R	R	R	R	R	CPN, ESBL and/or AmpC	blaKPC; blaSHV
MJH 617	Rectal swab	2018	R	R	I	R	R	R	R	R	R	S	S	R	R	S	R	R	R	CPN and/or ESBL	blaKPC; blaTEM
MJH 618	Urine	2018	R	R	I	R	R	R	R	R	R	R	R	R	R	R	R	R	R	CPN, ESBL and/or AmpC	blaKPC
MJH 619	Urine	2018	R	R	I	R	R	R	R	R	R	S	S	R	R	R	R	R	R	CPN and/or ESBL	blaKPC
MJH 620	Urine	2018	R	R	S	R	R	R	R	R	R	R	S	R	R	R	R	R	R	CPN, ESBL and/or AmpC	blaKPC
MJH 621	Rectal swab	2018	S	R	S	I	R	R	R	R	R	S	S	R	R	I	R	R	R	CPN and/or ESBL	blaKPC
MJH 622	Pus	2018	S	S	S	I	R	R	R	R	I	S	S	R	R	I	R	R	R	CPN and/or ESBL	blaSHV
